# RUNX transcription factors: biological functions and implications in cancer

**DOI:** 10.1007/s10238-023-01281-0

**Published:** 2024-03-02

**Authors:** Xinyi Chen, Lu Wang, Mu Yang, Weiheng Zhao, Jingyao Tu, Bo Liu, Xianglin Yuan

**Affiliations:** Department of Oncology, Tongji Hospital, Tongji Medical College, Huazhong University of Science and Technology, Jie Fang Road 1095, Wuhan, Hubei Province China

**Keywords:** RUNX family, Angiogenesis, Tumor cell stemness, Drug resistance, Tumor microenvironment, Signaling pathways

## Abstract

Runt-related transcription factors (RUNX) are a family of transcription factors that are essential for normal and malignant hematopoietic processes. Their most widely recognized role in malignancy is to promote the occurrence and development of acute myeloid leukemia. However, it is worth noting that during the last decade, studies of RUNX proteins in solid tumors have made considerable progress, suggesting that these proteins are directly involved in different stages of tumor development, including tumor initiation, progression, and invasion. RUNX proteins also play a role in tumor angiogenesis, the maintenance of tumor cell stemness, and resistance to antitumor drugs. These findings have led to the consideration of RUNX as a tumor biomarker. All RUNX proteins are involved in the occurrence and development of solid tumors, but the role of each RUNX protein in different tumors and the major signaling pathways involved are complicated by tumor heterogeneity and the interacting tumor microenvironment. Understanding how the dysregulation of RUNX in tumors affects normal biological processes is important to elucidate the molecular mechanisms by which RUNX affects malignant tumors.

## Introduction

RUNX proteins belong to a family of transcription factors. These proteins are master regulators of embryonic development and they play key regulatory roles in a wide range of biological processes, such as cell proliferation, apoptosis, differentiation, and lineage determination [1, 2]. In mammals, three different genes encode the three RUNX proteins, namely RUNX1, RUNX2, and RUNX3 [3]. The expression pattern of the RUNX family is highly dynamic, depending on the developmental stage and tissue microenvironment [4]. Functionally, RUNX1 is indispensable for the establishment of definitive hematopoiesis [5]. RUNX2 is considered to play a key role in osteogenic differentiation and bone formation [6]. RUNX3 acts as a tumor suppressor in gastric cancer, colon cancer, and some other solid tumors, but it is usually inactivated during tumor progression due to loss of heterozygosity, promoter hypermethylation, histone modification, and protein mislocalization [7]. All three RUNX proteins have a highly conserved DNA-binding domain, called the Runt domain, which heterodimerizes with the common non-DNA-binding core binding factor β (CBF-β) subunit. This interaction results in a structural change that replaces the repression domain and stabilizes the binding of RUNX proteins to their consensus motifs [8]. Deletion of any of the RUNX genes in mice results in lethality [9, 10], highlighting their fundamental and essential role in the process of development. The RUNX family is functionally related to major developmental pathways including the TGFβ signaling pathway [11], Wnt/β-catenin signaling pathway [12], Hedgehog signaling pathway [13–15], Notch signaling pathway [16], MAPK signaling pathway [17], and Hippo-YAP pathway [18].

RUNX1, encoded by the RUNX1 gene located on human chromosome 21, was first characterized in 1991. It is also known as acute myeloid leukemia 1 (AML1) because it is known to be involved in the t(8;21) chromosome translocation in patients with acute myeloid leukemia [19]. It is a transcription factor involved in hematopoietic processes [20] and is essential for the maturation of lymphocytes and megakaryocytes in adults [21]. Meanwhile, an increasing number of studies have revealed the pro- or anti-cancer roles of RUNX1 in solid tumors. Abnormal overexpression of RUNX1 has been observed in ovarian epithelial cancer [22], renal clear cell carcinoma [23], gastric cancer [24], colorectal cancer [25], and pancreatic cancer [26]. RUNX2, also known as core binding factor α1 (CBFα1), is the most specific marker gene in the early stages of bone formation, and plays a key role in the regulation of cell proliferation in osteoblasts and endothelial cells [27]. Similarly, several studies have shown that RUNX2 is also closely associated with the occurrence and development of tumors, such as breast cancer [28], colorectal cancer [29], thyroid cancer [30], and pancreatic cancer [31]. RUNX3 has been defined as both a tumor suppressor and a tumor promoter, and it can play such contradictory roles even in the same tumor, which may reflect the complex role of RUNX3 in tumorigenesis [32]. Compared with normal gastric epithelial cells, gastric cancer cells gradually lose RUNX3 expression as they gain high invasiveness with cancer progression. After the first study demonstrating that RUNX3 has a tumor suppressive role [33], an increasing studies have reached the same conclusion, suggesting that RUNX3 also plays a tumor suppressive role in solid tumors, such as colon cancer [34], lung cancer [35], breast cancer [36], glioma [37], renal cancer [38], and hepatocellular carcinoma [39].

Signaling pathways involving or dependent on RUNX play crucial roles in different processes of tumor progression, including tumor proliferation, metastasis, angiogenesis, tumor stemness, and chemoresistance. In this review, we aim to summarize and provide an overview of recent research on RUNX-mediated biological effects in tumors. With key examples, we will discuss how RUNX participates in different signaling pathways and biological processes to regulate proliferation and affect the progression of solid tumors.

## RUNX proteins in the landscape of cancer expression

### RUNX1 in carcinogenesis: a dual function

Among the RUNX family, RUNX1 exhibits a particularly complex role across different types of cancer. RUNX1 is one of the genes significantly mutated in luminal estrogen-receptor-positive (ER+) breast cancer. Its expression is lost during the development of ER+ breast cancer, suggesting the tumor-suppressive role of RUNX1 [40]. A follow-up study supported the idea that RUNX1 mainly acts as a tumor suppressor in ER+ breast cancer, and it can exert oncogenic effects by suppressing the estrogen-mediated inhibition of AXIN1 and activation of the Wnt/β-catenin signaling pathway [41]. In contrast, it has also been shown that RUNX1 levels are abnormally elevated in triple-negative breast cancer (TNBC) and this is associated with a poor prognosis, indicating that RUNX1 plays a pro-tumor role in TNBC [42]. An analysis of data from multiple databases confirmed the abnormally high expression levels of RUNX1 in cervical cancer [43]. However, research by Zhu et al. contradicts this by showing that RUNX1 can be downregulated in cervical cancer via miR-20a, thereby attenuating the cytotoxic effects of NK cells against cervical cancer cells [44]. The methylation level of the RUNX1 promoter is low in renal clear cell carcinoma, and the expression of RUNX1 is upregulated in renal clear cell carcinoma tissues compared with normal tissues [45]. Research by Janta et al. has confirmed that RUNX1 is aberrantly upregulated in prostate cancer and facilitates the EMT phenotype [46]. Elevated expression of RUNX1 has also been observed in glioblastoma (GBM) samples [47, 48]. Qiu et al. demonstrated that aberrant activation of the USP10/RUNX1 signaling axis in GBM maintains the mesenchymal properties of GBM cells, thereby promoting the progression of GBM [49]. Xu et al. substantiated that RUNX1 is markedly upregulated in GBM tissues, particularly in recurrent GBM tissues and in temozolomide-resistant GBM cells [50]. Intriguingly, in neuroblastoma, RUNX1 exhibits elevated expression levels in benign ganglioneuromas (GN) and well-differentiated tissues, while displaying reduced expression in poorly differentiated and undifferentiated tissues, suggesting its tumor-suppressive role in neuroblastoma [51]. Moreover, RUNX1 is also aberrantly upregulated in human pituitary tumors, contributing to tumor progression [52]. In an osteosarcoma study, the expression levels of RUNX1 mRNA and protein were found to be higher in tumor tissues than in normal tissues adjacent to the tumor [25]. Similarly, Jin et al. substantiated that RUNX1 is upregulated in oral squamous cell carcinoma (OSCC) tissues and cells, promoting cellular proliferation, adhesion, and migration while inhibiting apoptosis [53]. Complementing these findings, He et al. demonstrated an upregulation of RUNX1 expression in lung cancer, where it fosters cellular proliferation by binding to the promoter of tartrate-resistant acid phosphatase 5 (ACP5) [54]. Pertaining to digestive system malignancies, Liu et al. found that RUNX1 expression was significantly upregulated in human pancreatic cancer samples and they confirmed the role of RUNX1 in promoting pancreatic cancer cell proliferation [26]. Another study on pancreatic cancer showed consistent results, with quantitative polymerase chain reaction results indicating that the mRNA level of RUNX1 was significantly higher in human pancreatic cancer samples than in normal pancreatic tissues [55]. Pharmacological inhibition of RUNX1 can significantly suppress tumor growth in patient-derived organoids of primary pancreatic cancer [56]. In a study of gastric cancer, Mitsuda et al. demonstrated that elevated levels of RUNX1 in gastric cancer activated the ErbB2/HER2 signaling pathway by up-regulating SOS1, which served to promote the proliferation of gastric cancer cells [24]. However, the opposite conclusion has also been reached, namely, that RUNX1 is downregulated in gastric cancer tissues [57], suggesting a complex role of RUNX1 in the progression of this type of cancer. In hepatocellular carcinoma, elevated RUNX1 levels have been shown to upregulate COL4A1 expression, thereby activating the FAK-Src signaling pathway and promoting the proliferation, migration, and invasion of hepatocellular carcinoma cells [58]. In colorectal cancer, Zhou et al. showed that the high expression levels of LRG1 also resulted in abnormally high expression levels of RUNX1 [59]. Meanwhile, several studies have demonstrated that the abnormally high expression level of RUNX1 in colorectal cancer is closely associated with the occurrence of epithelial-mesenchymal transition (EMT) [25, 60]. To sum up, RUNX1 serves as a double-edged sword in cancer development, acting as either a tumor suppressor or a pro-tumor agent, depending on the type of cancer.

### RUNX2 in carcinogenesis: a predominant oncogenic contributor

RUNX2 expression is another key aspect of cancer pathology. In the realm of choroidal melanoma, Zhang et al. corroborated that RUNX2 is markedly upregulated and is directly targeted by METTL14 through N6-methyladenosine modification, contributing to its elevated expression [61]. This is in parallel with its overexpression in osteosarcoma, which has been linked to the downregulation of p53 and miR-34 [62]. Moreover, frequent amplification of the RUNX2 gene in osteosarcoma cell lines correlates with elevated RUNX2 levels, subsequently initiating MYC transcription and driving osteosarcoma tumorigenesis and progression [63]. Kim et al. affirmed the high expression levels of RUNX2 in osteosarcoma and identified it as a key transcription factor that sustains tumor cell survival, modulating a range of downstream target genes such as MYC through the induction of SOX9 and interactions with JMJD1C [64]. Research by Green et al. substantiated the upregulation of RUNX2 expression in tumors of patients with high-grade primary bone cancer [65]. In a parallel investigation, Onodera et al. scrutinized 137 cases of invasive ductal carcinoma of the breast through immunohistochemical staining and documented overexpression of RUNX2 [66]. Concurrently, elevated levels of RUNX2 in cervical cancer were found to be associated with decreased miR-218-5p expression, and this high expression of RUNX2 positively regulated cervical cancer cell proliferation [67].Wang et al. found that MRE11 plays a pro-cancer role in oral cancer through the RUNX2/CXCR4/AKT/FOXA2 signaling axis, and both MRE1 and RUNX2 have been shown to be highly expressed in oral cancer samples [68]. Sancisi et al. demonstrated that RUNX2 expression is reactivated in thyroid and breast cancers [69]. In epithelial ovarian cancer (EOC), RUNX2 promotes cell proliferation and invasion by regulating PKD2 and PKD3, thereby activating the MAPK/ERK1/2 signaling pathway, a finding that is further corroborated by Tong et al. who also confirmed elevated RUNX2 expression in EOC tissues and cells [70, 71]. Concurrently, both RUNX2 and MAPK11 are overexpressed in clear cell renal cell carcinoma (ccRCC) tissues and cell lines, enhancing the proliferation and migration of ccRCC cells [72]. In a study aligned with existing findings, Wu et al. revealed a marked upregulation of RUNX2 in ccRCC tissues. Mechanistically, the oncogenic capabilities of RUNX2 were attributed to its downregulation of the tumor suppressor NOLC1, which subsequently facilitated the growth and metastasis of ccRCC cells [73]. In pancreatic cancer, RUNX2 is also abnormally overexpressed, and its elevated expression is associated with the malignant behavior of the tumor, demonstrating significant diagnostic capability [74]. Guo et al. demonstrated the upregulation of RUNX2 expression in clinical samples of gastric cancer tissues and found that RUNX2 transcriptional activation of its downstream target, YAP1, promotes the progression of gastric cancer [75]. Moreover, upregulated RUNX2 in gastric cancer also promotes gastric cancer progression through transcriptional activation of MGAT5 and MMP13 [76]. RUNX2 is upregulated in gastric cancer, and in colorectal cancer patients, the expression levels of RUNX2 and MSN are significantly correlated, with both being overexpressed. MSN promotes colorectal cancer progression through the β-catenin-RUNX2 signaling axis [77]. Evidently, RUNX2 generally acts as a tumor facilitator, often collaborating with other signaling pathways to exacerbate cancer progression.

### RUNX3 in carcinogenesis: a primary tumor suppressor

Emphasis is placed on RUNX3, widely acknowledged as a tumor suppressor, and the implications of its downregulation in selected cancer types. For instance, a study by Zhou et al. found diminished expression of RUNX3 in OSCC specimens [78]. In cutaneous melanoma, RUNX3 acts as a tumor suppressor, with its expression being significantly downregulated in both primary and metastatic tumors [79]. In breast cancer, RUNX3 serves as a frequently inactivated or downregulated tumor suppressor that inhibits the proliferative and transformative potential of estrogen receptor α (ERα)-dependent cells, such as the MCF-7 cell line [36]. In a study by Bai et al., RUNX3 was demonstrated to be negatively regulated by overexpressed miR-20a-5p in TNBC, leading to a significant reduction in both its mRNA and protein levels [80]. A study by Paudel et al. examined the expression of RUNX3 in 100 cases of ovarian epithelial carcinoma (EOC) and 20 normal ovarian tissues, and the results suggested that RUNX3 expression is significantly elevated in EOC tissues [81]. Another study found that RUNX3 expression is lost in non-small cell lung cancer (NSCLC), leading to the upregulation of CCL5 and CCL19 in NSCLC cells, which was associated with tumor-associated bone destruction [82]. In addition, RUNX3 has been shown to destabilize the oncogenic protein MYC, thereby exerting a suppressive effect on gastrointestinal and lung cancers [83]. Zheng et al. demonstrated that RUNX3 expression is significantly down-regulated in renal cancer tissues, and that the loss of RUNX3 function in renal cancer tissues promotes the proliferation, migration, and invasion of renal cancer cells [84]. Complementing these findings, additional research has verified that RUNX3 expression is notably suppressed in metastatic renal cancer tissues due to hypermethylation of CpG islands [85]. Rehman et al. examined the expression of RUNX3 in 58 cases of esophageal cancer and matched adjacent normal tissues, and found that the expression level of RUNX3 mRNA was significantly increased in the tumor tissues from 31/57 esophageal cancer patients compared with its level in the corresponding normal tissues, suggesting that RUNX3 also plays a pro-cancer role [86]. However, the opposite conclusion has been made for esophageal squamous cell carcinoma (ESCC). Tonomoto et al. studied 61 ESCC clinical samples and found that methylation of the RUNX3 promoter region resulted in the absence of RUNX3 expression in tumor tissues [87]. Similarly, Horiguchi's research confirmed the downregulation of RUNX3 expression in pancreatic cancer [88]. The expression of RUNX3 is notably diminished in gallbladder cancer tissues and cells, largely attributed to DNA Methyltransferase 1 (DNMT1)-mediated methylation [89]. An analysis of 108 clinical samples of hepatocellular carcinoma showed that miR-106b-5p, which is upregulated in hepatocellular carcinoma, exerted a pro-cancer effect through the inhibition of RUNX3, and that the targeting of RUNX3 by miR-106b-5p resulted in its decreased expression in tumor tissues [90]. Concurrently, research conducted by Sakakura et al. identified a notable downregulation of RUNX3 in gastric cancer and its peritoneal metastases, primarily attributable to methylation in the RUNX3 gene's promoter region [91]. Likewise, in gastric cancer, Ju et al. confirmed that RUNX3 principally inhibits the Wnt signaling pathway through its interaction with the TCF4/β-catenin complex. Intriguingly, in certain gastric cancer cell lines such as KatoIII and SNU668, RUNX3 paradoxically elevated Wnt signaling activity, implying a cell-context-dependent role for RUNX3 [92]. Additionally, in gastric cancer, RUNX3 plays a role in suppressing cell proliferation and tumor growth, an effect mediated through the co-activation of the transcription factor Ets-1 by JMJD1A and the reduction in H3K9me1/2 levels [93]. Investigations in colorectal cancer confirmed that a decline in RUNX3 expression correlates with increased cell proliferation and invasion [94]. This was further corroborated by Wu et al., who detected a marked downregulation of RUNX3 in colorectal cancer, concomitant with an inverse correlation with HER2 expression [95]. Cumulatively, these findings underscore RUNX3's predominant function as a tumor suppressor, consistently found to be downregulated in diverse cancer types.

**Fig. 1:**
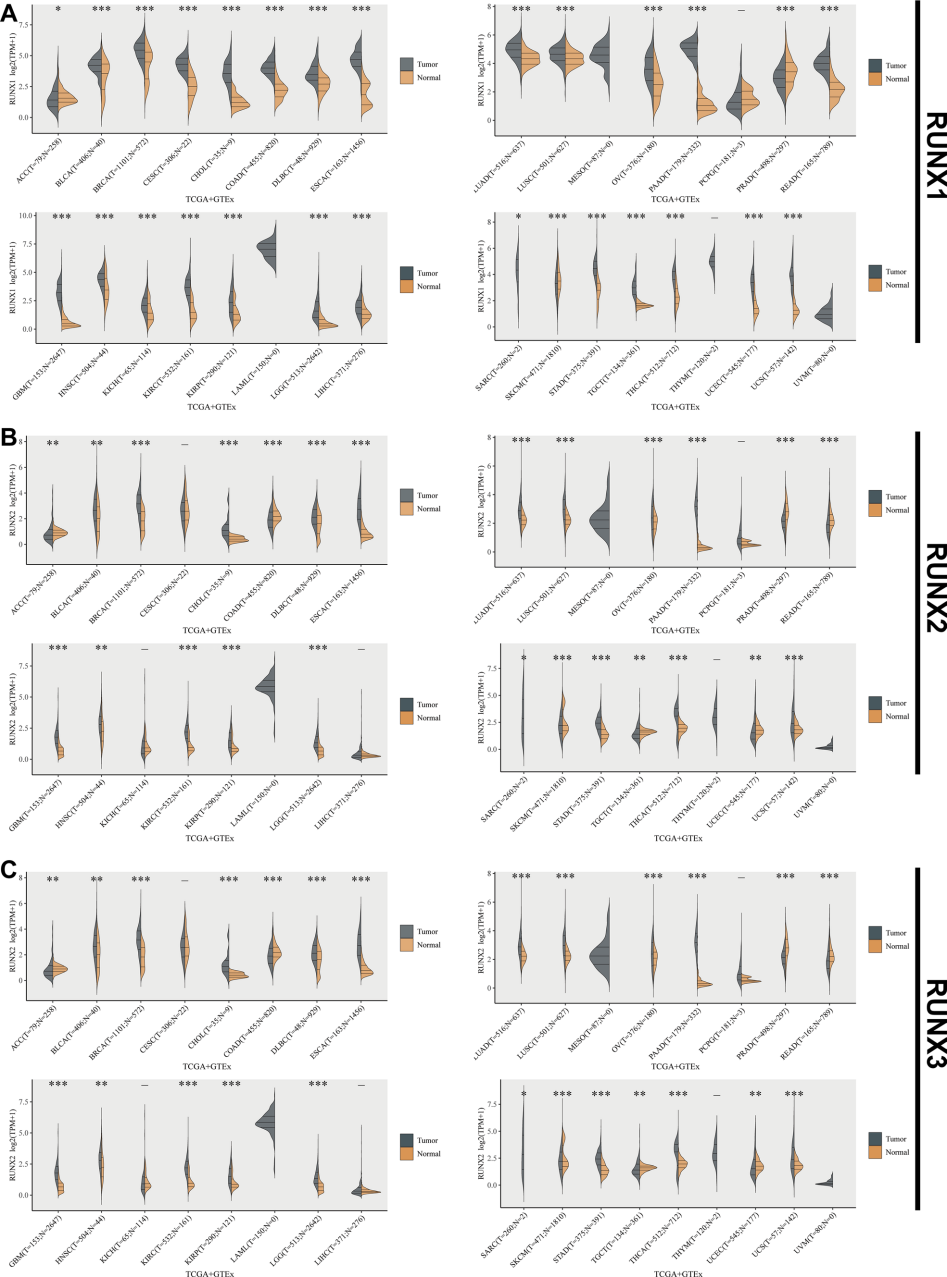
Pan-cancer assessment of RUNX family members' expression in comparison with normal tissues, sourced from the TCGA and GTEx databases. The violin plots consistently illustrate differences between normal tissues (depicted in orange) and tumor samples (depicted in gray). **A** RUNX1, **B** RUNX2, and **C** RUNX3 expression variations among different malignancies. *P < 0.05, **P < 0.01, ***P < 0.001

Further substantiating our discussion on the expression profiles of RUNX family proteins in tumor tissues, an analysis utilizing data from TCGA and GTEx databases provides additional insights into their pan-cancer expression patterns **(**Fig. 1**)**. Our comprehensive analysis of RUNX family expression across various cancer types further elucidates their role in tumorigenesis.

## Prognostic implications of RUNX proteins

Expanding on the aforementioned roles of RUNX proteins in tumorigenesis, this section focuses on their clinical utility as prognostic markers.

### RUNX1: a diverse prognostic indicator

RUNX1 is implicated in diverse prognostic outcomes across various cancer types. For instance, in patients with head and neck squamous cell carcinoma (HNSCC), elevated levels of RUNX1 are associated with more advanced disease stages, as indicated by American Joint Committee on Cancer staging, T-staging, and N-staging. Furthermore, multivariate Cox regression analyses have substantiated that elevated RUNX1 levels serve as an independent prognostic factor for poor overall survival (OS) in this patient population [96]. In TNBC patients, elevated levels of RUNX1 are associated with poor prognosis and have been established as an independent prognostic marker through multivariate Cox regression analysis [42]. However, certain studies have also reached the opposite conclusion, finding that the absence of RUNX1 expression in breast cancer is associated with activation of the TGF-β and WNT signaling pathways, and that a low RUNX1 expression level suggests a poor prognosis in breast cancer [97]. Moreover, diminished RUNX1 expression is associated with reduced OS in patients with NSCLC, serving as a predictive factor for adverse prognosis in this malignancy [98]. Research by Ramsey et al. substantiates that RUNX1 functions as a tumor suppressor in lung adenocarcinoma (LUAD), where its downregulation correlates with worse OS [99]. In high-grade serous ovarian cancer, RUNX1 stands as an independent prognostic marker for patient outcomes or therapeutic response [100]. Elevated expression levels of both RUNX1 and its downstream target, REXO2, in isocitrate dehydrogenase wild-type low-grade gliomas are indicative of unfavorable prognosis [101]. Zhang et al. performed an online database analysis and demonstrated that RUNX1 is an independent prognostic factor in low-grade gliomas, and that it may target interferon-γ receptor 2 (IFNGR2) to regulate the proliferation, invasion, and migration of glioma cells. Zhang et al. also confirmed that glioma patients with high RUNX1 expression have a significantly lower survival rate compared to those with low RUNX1 expression [48]. Additionally, patients with low-grade gliomas with high expression levels of RUNX1 and/or IFNGR2 have a worse prognosis, with a significant increase in the infiltration of M2 macrophages [102]. Abnormally high expression levels of RUNX1 are associated with poorer OS in patients with ccRCC [45]. Similarly, Rooney et al. confirmed that RUNX1 acts as an oncogenic driver in ccRCC, associating elevated RUNX1 expression with significantly poorer clinical outcomes compared to lower expression levels [103]. In a study of pancreatic cancer, the results of Kaplan–Meier survival analysis based on immunohistochemistry score data for RUNX1 suggested that a high expression level of RUNX1 is associated with a shorter OS time [55]. These findings underscore the context-dependent role of RUNX1 as a prognostic marker.

### RUNX2: generally a poor prognostic marker

Elevated levels of RUNX2 consistently serve as an adverse prognostic marker across multiple cancer types. For instance, in cervical cancer, high RUNX2 expression correlates with poor prognosis, and both RUNX2 and its inhibitory counterpart, miR-218-5p, are identified as potential prognostic markers [67]. Research by Li et al. indicated that the upregulation of RUNX2 in EOC is likely associated with tumor progression and unfavorable outcomes [104]. Similarly, heightened expression of RUNX2 is indicative of a poor prognosis in breast cancer patients [105]. Zhang et al. also confirmed that abnormal overexpression of RUNX2 in breast cancer correlates with advanced TNM stages, metastasis, and unfavorable prognosis [106]. Elevated levels of Parathyroid hormone-like hormone (PTHLH), an autocrine/paracrine ligand in HNSCC, not only serve as a marker of poor prognosis but also exhibit a significant positive correlation with RUNX2 expression, which, in conjunction with the RUNX2-PTHLH signaling axis, contributes to HNSCC progression [107]. Overexpression of RUNX2 is significantly associated with poor survival in patients with ccRCC [73]. Liu et al. revealed that aberrant overexpression of RUNX2 in bladder urothelial carcinoma (BLCA) is indicative of both high infiltration of cancer-associated fibroblasts (CAFs) and poor prognosis in BLCA patients [108]. Notably, in prostate cancer, particularly under conditions of bone metastasis, RUNX2 expression is significantly upregulated [109]. In hepatocellular carcinoma, elevated RUNX2 expression is likewise associated with shorter survival times [110]. Similarly, research by Guo et al. corroborated that RUNX2 is highly expressed in the early stages of gastric cancer and is positively correlated with unfavorable clinical outcomes [75]. In another gastric cancer study, RUNX2 was found to promote metastasis through the upregulation of COL1A1 expression, with patients displaying elevated levels of both RUNX2 and COL1A1 experiencing reduced survival times, thereby indicating a poor prognosis [111]. Complementing these findings, a study by Yi et al. significantly correlated elevated RUNX2 expression levels with metastatic progression and poor survival rates in patients with colon cancer [112]. Overall, RUNX2 is commonly associated with poor survival and could serve as an independent prognostic marker in multiple types of cancer.

### RUNX3: primarily a tumor suppressor with prognostic implications

RUNX3, predominantly recognized as a tumor suppressor, serves as a crucial prognostic marker, with its downregulation often indicative of adverse prognoses. For instance, in papillary thyroid cancer (PTC), hypermethylation at specific CpG sites leading to downregulated RUNX3 expression has been significantly associated with an elevated risk of tumor recurrence [113]. In neuroblastoma clinical samples, research conducted by Yu et al. corroborated that patients with low RUNX3 expression exhibited significantly reduced survival rates, whereas higher levels of RUNX3 expression were frequently observed in patients at favorable stages 1 and 2 [114]. In EOC, Heinze et al. substantiated that methylation of RUNX3 is correlated with patients' progression-free survival (PFS) and OS, indicating that a combination of RUNX3 and CAMK2N1 methylation serves as an independent prognostic marker [115]. In GBM, LMTK2 mediates tumor suppression by upregulating RUNX3, which in turn inhibits the Notch signaling pathway; low levels of LMTK2 are associated with poor overall survival, thereby suggesting that both LMTK2 and RUNX3 collectively influence the prognosis of GBM patients [116]. Kitago et al. confirmed that downregulation of RUNX3 in melanoma is indicative of poor prognosis for patients [79]. Moreover, low expression of RUNX3 in OSCC tissues is associated with inferior 5-year overall survival rates [78]. In NSCLC, Yu et al. tested clinical samples and found that methylation of the RUNX3 promoter led to its reduced or absent expression, suggesting a poor prognosis [117]. The down-regulation of RUNX3 expression and its loss of function in renal cancer tissues are closely related to a poor prognosis of patients with renal cancer [84]. Cai et al. demonstrated that RUNX3 expression is down-regulated in gallbladder cancer due to DNMT1-mediated promoter hypermethylation, and its downregulation is associated with a poor prognosis of patients with gallbladder cancer [89]. In a study of ESCC, the results of clinical sample analysis suggested that a low expression level of RUNX3 is closely associated with more advanced T-staging and the occurrence of lymph node metastasis, and that inactivation of RUNX3 leads to a poor prognosis for patients with ESCC [118]. Similarly, research conducted by Fujimoto et al. has demonstrated that in pancreatic cancer, downregulation of RUNX3 expression and its subsequent methylation serve as negative prognostic indicators, especially when combined with CA19-9 levels, enhancing the sensitivity for detecting early-stage pancreatic cancer [119]. Research by Horiguchi et al. corroborated a significant downregulation of RUNX3 in pancreatic cancer, with median survival durations for patients exhibiting normal and reduced RUNX3 expression being 1006 and 643 days, respectively, thereby establishing the negative prognostic impact of RUNX3 downregulation [88]. Ning et al. disclosed that reduced JMJD1A expression in gastric cancer is associated with invasive phenotypes and poor prognosis, and this association is further substantiated by a positive correlation between JMJD1A and RUNX3 expression, indicating that reduced RUNX3 expression serves as an indicator of unfavorable prognosis [93]. In colon cancer, increased RUNX3 expression levels in tumor epithelial cells and stromal cells are independent predictors of a good prognosis [120]. Complementing these findings, Zhang et al. confirmed that decreased expression of RUNX3 in CRC tissues and cells is linked to poor prognosis, accentuating its function as a tumor suppressor [94]. As the evidence suggests, low expression levels of RUNX3 are generally associated with a poor prognosis, highlighting its role as a tumor suppressor.

**Fig. 2:**
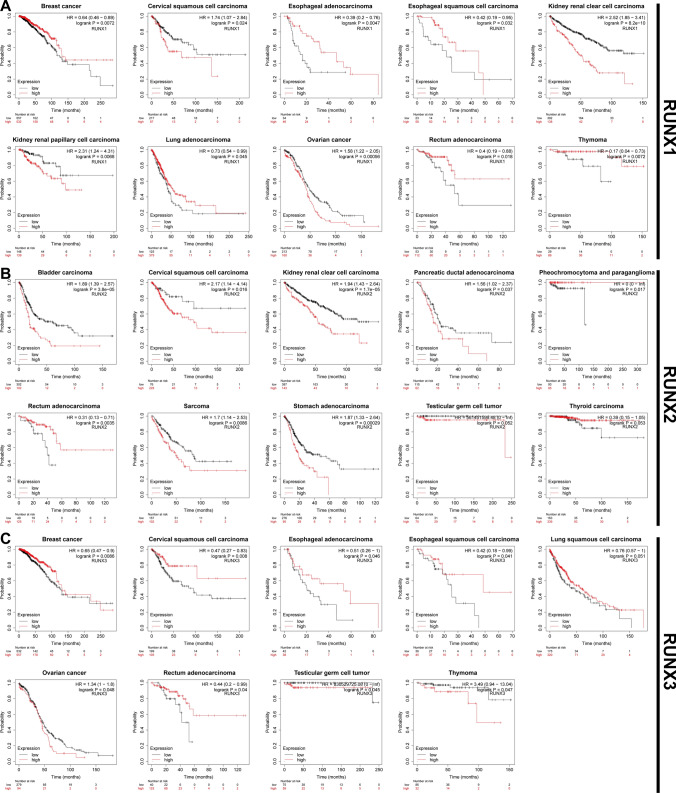
Prognostic implications of RUNX family gene expressions in various cancers.** A** RUNX1, **B** RUNX2, and **C** RUNX3: Kaplan–Meier curves depict the correlation of high RUNX expression (in red) with overall survival (OS). Data sourced from the Kaplan–Meier plotter database. *P*-values and hazard ratios (HR) were calculated using the logrank test to indicate the statistical significance of the survival outcomes

Moreover, an assessment of the association between RUNX family genes and OS in multiple tumor types was conducted using the Kaplan–Meier plotter online database (Fig. 2). These database findings corroborate the dual prognostic implications of RUNX1, underline the primary negative prognostic influence of RUNX2, and validate the tumor-suppressive role of RUNX3. Collectively, these data provide substantial evidence for the integral association between RUNX family genes and tumor prognostic outcomes, further solidifying their clinical utility as prognostic markers.

## RUNX family proteins and tumor stemness

### RUNX1: multifaceted influences on stem cell properties

RUNX1 plays a significant role in the regulation of tumor stemness, warranting closer examination. RUNX1 critically influences the stem-like properties of cancer cells, with evidence pointing to its role in the stabilization of leukemia stem cell attributes in a pluripotent model [121]. Research by Jain et al. demonstrated that RUNX1 potentially promoted stem cell activation in hair follicle stem cells and skin and oral squamous cell carcinoma through the regulation of lipid metabolism and its impact on the Wnt signaling pathway [122]. In glioblastoma stem cells (GSCs), research by Santoni et al. demonstrated overexpression of RUNX1 splice variants Aml1b and Aml1c during GSC differentiation [123]. In breast cancer, RUNX1 critically influences both the EMT and stemness, both of which are robustly linked to invasive tumor characteristics [124]. A study by Fernandez et al. corroborated the observation that elevated RUNX1 expression predominantly facilitates the manifestation of cancer stem cell (CSC) markers in TNBC [125]. Conversely, some reports offer contrasting perspectives. For instance, Hong et al. observed that RUNX1 inhibits stem cell activities in breast cancer, consequently restraining tumor progression [126]. Additional evidence by Kulkarni et al. suggested that RUNX1, in conjunction with RUNX3, curtails the expression of YAP, thereby mitigating YAP-induced EMT and stemness [127]. Similarly, Chimge et al. disclosed that in ER + breast cancer, the lack of RUNX1 triggers an increased expression of stem cell markers [41]. Fritz et al. elucidated that RUNX1 and RUNX2 have divergent effects on breast cancer stem cells; specifically, the downregulation of RUNX1 accompanied by RUNX2 upregulation fosters EMT and sustains stem cell-like properties [128]. Overall, the data indicates that RUNX1's regulatory role in tumor stemness varies in a cancer subtype-specific manner.

### RUNX2: predominantly a promoter of cancer stem cell traits

RUNX2 exerts a pivotal influence on the regulation of tumor stemness across a diverse array of cancer subtypes. Zhang et al. showed that RUNX2 promotes the stem cell properties of CD44+/CD24− breast cancer, while miR-205 reverses the stemness by inhibiting RUNX2 [129]. Moreover, elevated RUNX2 expression in breast cancer has been linked to enhanced tumor stem cell characteristics, thereby facilitating breast cancer cell metastasis [106]. Similarly, Yin et al. corroborated that in breast cancer, RUNX2 promotes the tumor stem cell phenotype through the recruitment of the NuRD(MTA1)/CRL4B complex [130]. Further substantiating the role of RUNX2 in breast cancer, Knutson et al. revealed that RUNX2 is instrumental in maintaining tumor stem cell activity, a mechanism intricately connected with phospho-progesterone receptors and EGF signaling pathways [131]. In LUAD, a cigarette extract was found to promote the expression of RUNX2, which then induced the upregulation of stemness markers in airway epithelial cells (AECs), leading to increased migration, invasion, and tumorsphere formation by tumor stem cells at the molecular level in AECs [132]. Senbanjo et al. demonstrated that CD44 regulates RUNX2 expression in prostate cancer, and that the interaction between RUNX2 and CD44 promotes the expression of metastasis-associated genes, such as osteopontin (OPN) and MMP-9, which in turn promotes the migration and invasion of prostate cancer cells [133]. In colorectal cancer, Yan et al. demonstrated that RUNX2 induces a stem cell phenotype in colon cancer cells by binding to BRG1 as a tight complex, thereby upregulating the transcription and expression of CD44, and promoting the invasion and migration of colon cancer cells [134]. Overall, evidence predominantly supports RUNX2's role in enhancing stem cell-like characteristics in a range of cancers, which is often linked to worse patient outcomes.

### RUNX3: mainly a negative modulator of tumor stemness

RUNX3 engages in intricate regulatory mechanisms governing tumor stemness, generally aligning with the prevailing notion that it serves as a suppressive modulator. In line with its recognized role as a suppressive modulator of tumor stemness, Jiang et al. substantiated that in LUAD, RUNX3 is directly downregulated by miR-1275, resulting in the activation of Wnt/β-catenin and Notch signaling pathways; this mechanism consequently enhances the stem-like properties of LUAD cells, thereby promoting tumorigenesis, recurrence, and metastasis [135]. Further research has indicated its negative regulation of the TEAD-YAP oncogenic complex, thereby reversing EMT and stem-like phenotypes in tumor cells, particularly in gastric cancer [136]. Voon et al. also demonstrated that if RUNX3 expression is absent in gastric cancer, it is prone to spontaneous EMT and aberrant TGF-β and Wnt signaling, which leads to an increase in a subpopulation of tumor cells with stem-cell-like properties [137]. Moreover, the deficiency of RUNX3 in murine gastric epithelial cells (GIF-14) is associated with enhanced stem-cell-like characteristics [138]. Balinth et al. showed that EZH2 inhibits the tumor suppressor RUNX3, which activates SETDB1 and ΔNp63α, driving an invasive tumor stem cell phenotype, and that the use of an EZH2 inhibitor reactivates RUNX3, thereby reversing this process [139]. In colorectal cancer, RUNX3 suppresses the stem cell phenotype of colorectal cancer cells by inhibiting Hedgehog signaling [13]. Overall, RUNX3 predominantly acts as a dampener of tumor stemness, distinguishing it from RUNX1 and RUNX2.

## RUNX proteins and angiogenesis

### RUNX1: multifaceted roles in angiogenesis

Angiogenesis, the formation of new blood vessels from pre-existing ones, plays a pivotal role in the progression and metastasis of tumors [140]. Within this complex biological process, the transcription factor RUNX1 has emerged as a multifaceted regulator, exhibiting both pro-angiogenic and anti-angiogenic activities depending on the cancer type. RUNX1 promotes angiogenesis by downregulating insulin-like growth factor binding protein-3 (IGFBP-3) [141]. In GBM, knockdown of RUNX1 in U-87 MG cells inhibits the angiogenesis of human umbilical vein endothelial cells, and a p38 MAPK inhibitor (SB203580) reduces RUNX1 expression levels; thus, RUNX1 may promote angiogenesis in gliomas through activation of the p38 MAPK signaling pathway [17]. However, there are also studies suggesting the opposite. For example, Liu et al. demonstrated that RUNX1 exerts an inhibitory effect on vascular endothelial growth factor (VEGF) A in hepatocellular carcinoma, hindering angiogenesis and thus, inhibiting the progression of hepatocellular carcinoma [142]. Similarly, Hong et al. demonstrated that RUNX1 inhibits angiogenesis and promotes apoptosis in neuroblastoma, thus preventing its progression [51]. Rada et al. found that activation of the RUNX1-Ang1 pathway was responsible for the high level of neutrophil infiltration through vessel co-opting in colorectal cancer liver metastases, and that high levels of neutrophil infiltration is a potential factor promoting the development of liver metastases [143]. Thus, RUNX1 emerges as a complex regulator of angiogenesis, with both pro-angiogenic and anti-angiogenic roles varying by cancer type.

### RUNX2: a key facilitator of angiogenesis and tumor progression

RUNX2's significance is underscored by its involvement in multiple physiological and pathological processes, including angiogenesis. Angiogenesis and bone formation are closely related processes, and it has been shown that RUNX2 overexpression in mesenchymal cells upregulates the expression of hypoxia-inducible factor 1α and VEGF, which stimulates angiogenesis [144]. Additional evidence supporting this role includes research indicating that RUNX2 mediates vascular formation in endothelial cells via glucose-activated phosphorylation [145]. Complementing these observations, a study by Papachristou et al. affirmed that RUNX2 critically contributes to the malignant transformation and progression of chondrosarcoma through the upregulation of VEGF [146]. Corroborating these findings, research by Cecconi et al. established that the Runt domain of RUNX2 plays an indispensable role in neoangiogenesis in melanoma, serving as a potent promoter of new blood vessel formation [147]. Similarly, a study by Niu et al. demonstrated that elevated RUNX2 levels in thyroid carcinoma induce the expression of vasculogenic factors VEGFA and VEGFC, thus promoting tumor invasiveness [148]. Furthermore, the angiogenesis-inhibiting effects of emodin in breast cancer cells may be attributable to the downregulation of RUNX2 transcriptional activity [149]. In neuroblastoma, extracellular matrix stiffness controls VEGF_165_ secretion through the YAP/RUNX2/SRSF1 axis and regulates tumor angiogenesis [150]. In hepatocellular carcinoma, Cao et al. have substantiated that elevated RUNX2 expression is implicated in the promotion of vasculogenic mimicry (VM), thereby facilitating tumor progression [110]. Thus, RUNX2 not only plays a role in angiogenesis but also influences tumorigenic processes related to vascular growth.

### RUNX3: primarily an inhibitor in angiogenic regulation

Attention is shifted to RUNX3, another key member of the RUNX family, commonly acknowledged as a tumor suppressor, to explore its potential role in the regulation of angiogenesis. Research by Chen et al. substantiates that RUNX3 serves as a tumor suppressor in prostate cancer by diminishing the secretion of VEGF, thereby inhibiting tumoral angiogenesis [151]. Complementing these findings, RUNX3 is observed to down-regulate VEGF expression in gastric cancer cells, thereby limiting angiogenesis and impeding tumor growth and metastasis [152]. In a complementary vein, Lee et al. have confirmed that RUNX3 serves as an inhibitor of HIF-1α in gastric cancer cells, promoting the prolyl hydroxylation and degradation of HIF-1α through interactions with PHD-2, ultimately suppressing hypoxia-induced angiogenesis within the tumor microenvironment [153]. Similarly, Kim et al. revealed that in colorectal cancer, RUNX3 curtails VEGF secretion, thereby obstructing endothelial cell proliferation and angiogenesis [154]. In oral squamous cell carcinoma, RUNX3 also inhibits VEGF activity and exerts anti-cancer effects [78]. However, divergent results do exist; for example, in EOC, a distinct transcriptional variant of RUNX3 appears to promote angiogenesis, thus functioning in a pro-oncogenic manner [155]. Taken together, the prevailing evidence predominantly supports the role of RUNX3 as a key inhibitor of angiogenesis.

## RUNX family and the intricate landscape of tumor metastasis

### RUNX1: the multifaceted orchestrator of metastasis

RUNX1 is a pivotal transcription factor implicated in the regulation of metastasis across various cancer types. Specifically, RUNX1 interacts with SNORA71C to accelerate breast cancer progression and metastasis [156]. Browne et al., utilizing the MMTV-PyMT transgenic mouse model, demonstrated that RUNX1 not only fosters tumor invasion and metastasis in breast cancer but also revealed its heightened expression in distal lung metastatic lesions [157]. Meanwhile, Liu et al. elucidated that RUNX1 augments the MAPK signaling cascade in HNSCC by directly engaging with the promoter region of OPN, thereby facilitating HNSCC metastasis [158]. In cervical cancer, the RUNX1 expression level is abnormally elevated, promoting EMT and significantly enhancing the invasion and metastasis of cervical cancer cells [43]. Doll et al. demonstrated that, in endometrial carcinoma, RUNX1 collaborates with an array of proteins such as CBFβ and members of the Ets transcription factor family to expedite distant metastasis, particularly to the lungs and para-aortic lymph nodes [159]. RUNX1 plays a role in promoting tumor metastasis in EOC, and Keita et al. demonstrated that RUNX1 is hypomethylated in tumor tissues with omental metastases [22]. Abnormally elevated RUNX1 levels in prostate cancer promote the EMT phenotype and activate the Akt/P38/JNK-MAPK signaling pathway driving the invasion and metastasis of prostate cancer cells [46]. In hepatocellular carcinoma, RUNX1 induces tumor cell migration, invasion, and metastasis by activating the COL4A1/FAK/Src signaling axis [160]. Of particular concern is that, in colorectal cancer, RUNX1 activates the TGF-β signaling pathway, which plays a dominant role in the EMT process of colorectal cancer cells. Therefore, up-regulation of this signaling pathway by RUNX1 can promote colorectal cancer cell migration and invasion [60]. Additionally, RUNX1 enhances liver metastasis of colorectal cancer by activating vessel co-option through ARP2/3 [161]. Meanwhile, it has been found that RUNX1 expression is up-regulated in colorectal cancer tissues and this promotes colorectal cancer metastasis by activating the Wnt/β-catenin signaling pathway and EMT [25]. RUNX1 also directly binds to the RNCR3 promoter region to transcriptionally upregulate RNCR3 expression. Moreover, RNCR3 overexpression blocks the inhibitory effect of miR-1301-3p on the proliferation and invasion of colorectal cancer cells, while upregulating AKT1 to promote colorectal cancer progression [162].Overall, RUNX1 plays a significant role in modulating metastasis through its interactions with multiple signaling pathways, thus representing a complex but crucial factor in the progression of diverse malignancies.

### RUNX2: predominantly a promoter of cancer metastasis

The seemingly paradoxical relationship between osteogenesis and metastasis finds a nexus in RUNX2, a transcription factor predominantly expressed in mesenchymal cells with an osteoblastic phenotype. Essential for bone formation, RUNX2 aberrantly overexpresses in specific tumor cells of breast and prostate origin, which eventually manifest invasive bone metastases. Such aberrant overexpression has been elucidated to bear a significant association with bone metastases [163]. In primary bone cancer, RUNX2 is aberrantly overexpressed and physically interacts with YBX1, thereby exerting pro-metastatic effects [65]. In osteosarcoma, research by Villanueva et al. substantiated that RUNX2 activates the OPN/SPP1 gene, consequently enhancing adhesion between osteosarcoma cells and pulmonary microvascular endothelial cells, which ultimately drives lung metastasis [62]. In thyroid cancer, RUNX2 promotes EMT and tumor invasion by inducing the expression of EMT-related molecules such as SNAI2, SNAI3, TWIST1, and MMP2 [148]. Wang et al. have corroborated that, in oral cancer, RUNX2 advances the EMT phenotype and metastasis through its synergistic interactions with CXCR4, AKT, and FOXA2 [68]. In conjunction with this, research by Yi et al. positions RUNX2 as an epigenetic orchestrator instrumental in facilitating EMT, hence suggesting its utility as a potential prognostic biomarker for breast cancer metastasis [112]. Additionally, in breast cancer, RUNX2 recruits the NuRD(MTA1)/CRL4B complex to catalyze histone deacetylation and ubiquitination, affecting a cohort of key genes including PPARα and SOD2, which play pivotal roles in promoting EMT and metastasis [130]. Li et al. identified ITGA5 as a novel transcriptional target of RUNX2 and demonstrated that RUNX2 fosters the recruitment and colonization of breast cancer cells in bone via ITGA5-dependent mechanisms, culminating in bone metastasis [164]. Complementing these findings, research by Sancisi et al. underscores that RUNX2 facilitates tumor metastasis in both thyroid and breast cancer, modulated through the synergistic control of BRD4 and c-JUN [69]. In LUAD, RUNX2 functions as a critical transcription factor that augments tumor cell EMT, migration, and invasion through the upregulation of the galectin-3 pathway and ROS activation [132]. In ccRCC, RUNX2 is up-regulated by ZIC2 and it enhances the proliferation and migration of ccRCC cells by transcriptionally suppressing the tumor suppressor, NOLC1, and dysregulation of ZIC2/RUNX2/NOLC1 signaling promotes ccRCC metastasis [73]. In BLCA, aberrantly overexpressed RUNX2 contributes to tumor metastasis by inducing an EMT phenotype [108]. Zou et al. showed that exosomal miR-1275 secreted by prostate cancer cells activates the SIRT2/RUNX2 signaling pathway to promote the proliferation and activity of osteoblasts, promoting the metastasis of prostate cancer [165]. In highly metastatic prostate cancer cells, RUNX2 is aberrantly overexpressed, a finding corroborated by the study conducted by Akech et al. [166]. RUNX2 phosphorylation plays a crucial role in the occurrence and development of prostate cancer, inducing tumor cells to develop an invasive phenotype, which ultimately contributes to their metastasis [167]. Corroborating these observations, Roy et al. affirmed that RUNX2 serves as a key gene promoting bone metastasis in prostate cancer by activating the MEK/ERK1/2 signaling pathway [168]. In a complementary study, Senbanjo et al. elucidated that within PC3 prostate cancer cells, RUNX2 forms a co-transcriptional complex with CD44-ICD, resulting in the upregulation of metastasis-associated genes and thereby promoting cellular invasion and migration [133]. Complementing the data in prostate cancer, Li et al. revealed that RUNX2 enhances metastasis in gastric cancer by upregulating COL1A1 expression [111]. In line with these findings, Cao et al. demonstrated that elevated RUNX2 expression in hepatocellular carcinoma facilitates EMT as well as tumor cell migration and invasion [110]. In summary, RUNX2 is not merely a bridge between bone formation and metastasis; it serves as a critical player in the metastatic pathways of several types of malignancies.

### RUNX3: a potential inhibitor of metastasis

Contrary to other RUNX family members predominantly implicated in the enhancement of metastasis, RUNX3 manifests an opposing role. Research conducted by Wang et al. corroborated RUNX3's tumor-suppressive role in melanoma, particularly in inhibiting cell migration and metastasis [169]. In renal cell carcinoma, Zheng et al. demonstrated that the downregulation of both RUNX3 and TGF-β in metastatic tissues, attributed to hypermethylation of CpG islands, is significantly associated with metastatic propensity and can be reversed by the application of a methylation inhibitor [85]. In prostate cancer, RUNX3 serves as a tumor suppressor. Its overexpression leads to the upregulation of TIMP-2, which in turn inhibits the expression and activity of MMP-2, thereby suppressing the metastasis of prostate cancer [151]. In esophageal squamous cell carcinoma (ESCC), overexpression of RUNX3 remarkably suppresses the phosphorylation of Smad2/3. Through the TGF-β/Smad signaling pathway, RUNX3 reverses EMT, subsequently inhibiting the invasion and metastasis of ESCC cells [118]. Notably, corroborating research by Whittle et al. elucidates that RUNX3 exhibits a bifunctional role in pancreatic ductal adenocarcinoma by simultaneously constraining cell proliferation and facilitating cellular migration and invasion, a mechanism intricately associated with Dpc4 (Smad4) status [170]. In cases of gastric cancer, however, the absence of RUNX3 accelerates the progression toward peritoneal metastasis [91]. Interestingly, in colorectal cancer, Zhang et al. demonstrated that hypermethylation-induced downregulation of RUNX3 disrupts the circMETTL3/miR-107/PER3 axis, thereby facilitating cancer metastasis [94]. Unlike its counterparts, RUNX3 mainly demonstrates an inhibitory function on metastasis, although the specifics can be context-dependent, highlighting the complex role it plays in the realm of metastasis.

## RUNX proteins and drug resistance in tumor therapy

### RUNX1's regulatory influence in drug resistance

Building upon RUNX1's involvement in angiogenesis and metastasis, it is crucial to explore its role in drug resistance. Fernández et al. demonstrated that in TNBC, RUNX1 binds to the androgen receptor (AR), leading to resistance to AR inhibitors in patients with TNBC [125]. In ovarian cancer, RUNX1 negatively regulates the expression of the miR-17-92 cluster, which leads to the upregulation of BCL2, the direct target of miR-17-92, resulting in significant inhibition of cisplatin-induced apoptosis, which may be associated with cisplatin resistance [171]. Hyperactivation of the RUNX1/IL-34/CSF-1R signaling axis is associated with the resistance of melanoma to BRAF-V600E inhibitors [172]. Wang et al. demonstrated that RUNX1 negatively regulates miR-101 expression in lung cancer cells, thereby hindering the sensitizing effect of miR-101 on cisplatin in lung cancer chemotherapy [173]. Xu et al. demonstrated that RUNX1 plays an oncogenic role in GBM, and that RUNX1 induces temozolomide resistance in GBM by up-regulating MRP1, which is negatively regulated by miR-128-3p [50]. In EOC, RUNX1 synergistically binds to the promoter region of insulin-like growth factor 1 receptor (IGF1R) with FOXO3a, contributing to the up-regulation of IGF1R expression, which can lead to the development of platinum-paclitaxel resistance in EOC [174]. Han et al. demonstrated the potential of employing RUNX1 as a biomarker of reference in devising chemotherapy regimens for patients diagnosed with gastric cancer [175]. In colorectal cancer, RUNX1 is a biomarker for the development of chemotherapy programs and it can activate the Hedgehog signaling pathway by up-regulating the expression of ABCG2, inducing resistance to 5-fluorouracil by colorectal tumor cells [15]. The data collectively suggests that RUNX1 acts as a key regulatory node in the establishment of drug resistance across diverse types of cancer, thereby offering multiple therapeutic avenues for intervention.

### RUNX2: mediator of chemoresistance

RUNX2 contributes to the chemo-resistant phenotype in several cancers. In TNBC, RUNX2 leads to chemoresistance in breast cancer cells through transcriptional activation of the target gene, MMP1 [28]. An analysis of osteosarcoma-related gene expression indicates that overexpression of RUNX2 can be a potential biomarker for chemotherapy failure in patients with osteosarcoma [176]. Similarly, research by Ozaki et al. demonstrated that RUNX2 attenuates cellular sensitivity to Adriamycin chemotherapy in human osteosarcoma by inhibiting the transcriptional activity of TAp73, a molecule involved in DNA damage response. This mechanism contributes to chemoresistance, and its disruption through RUNX2 knockdown enhances Adriamycin sensitivity while upregulating TAp73 and its target genes [177]. In related research, the same team also revealed that RUNX2 inhibits the transcriptional and pro-apoptotic activities of p53 through functional collaboration with HDAC6 in human osteosarcoma, potentially implicating a role for RUNX2 in Adriamycin resistance in this cancer type [178]. Sugimoto et al. demonstrated that RUNX2 confers gemcitabine resistance in pancreatic cancer AsPC-1 cells through the inhibition of TAp63, suggesting that targeting RUNX2 may serve as a novel strategy to enhance the efficacy of gemcitabine treatment in p53-deficient pancreatic tumors [179]. RUNX2 has also been shown to be significantly overexpressed in platinum-chemotherapy-resistant gastric cancer cells and tissues, and RUNX2 reduces the response of gastric cancer to chemotherapeutic drugs by negatively regulating p53-mediated apoptosis [180]. These findings position RUNX2 as a significant actor in the development of chemoresistance and a possible target for improving the efficacy of existing treatments.

### RUNX3: a tumor-suppressive regulator in cancer drug resistance

Kim et al. demonstrated that re-expression (activation) of RUNX3 enhances the susceptibility of NSCLC to Sc-conjugated cetuximab, and that clinical efficacy can be improved through the combined use of therapeutics with RUNX3 activity [181]. Barghout et al. showed that RUNX3 expression was elevated in the tumor tissues of patients with carboplatin-resistant EOC compared to those with carboplatin-sensitive EOC, suggesting that a high RUNX3 expression level contributes to the development of chemoresistance in EOC [182]. In pancreatic cancer, loss of RUNX3 expression leads to the upregulation of multidrug resistance proteins (MRP), consequently increasing resistance to gemcitabine and adversely affecting patient prognosis [88]. Tan et al. found that in hepatocellular carcinoma, HCV core protein reduces sensitivity to cisplatin by downregulating RUNX3 via inhibition of NR4A1 and upregulation of Smad7 [183]. In gastric cancer, RUNX3 is targeted and suppressed by miR-106a, particularly in multidrug-resistant (MDR) cell lines. This downregulation facilitates the efflux of anthracycline drugs (ADR) and inhibits drug-induced apoptosis, thereby advancing mechanisms of multidrug resistance and chemoresistance [184]. Collectively, these findings underscore RUNX3's role as a tumor-suppressive gene in mediating drug resistance, highlighting its context-dependent impact across various cancer types and therapeutic approaches, thereby deepening our understanding of the RUNX family's tumor-suppressive influence on drug resistance.

## Summary and perspectives

RUNX transcription factors function as pivotal developmental regulators, indispensable for cellular differentiation across diverse tissue types. These proteins, despite recognizing the same DNA sequences, have unique C-terminal structural domains that lead to varying target binding, occasionally yielding contradictory outcomes. Depending on the cellular context, RUNX transcription factors may transition between roles as tumor suppressors and oncogenes. Intricate interplay exists among the various members of the RUNX family, with this interplay largely dependent on the relative expression levels of each family member in different tissues.

**Fig. 3:**
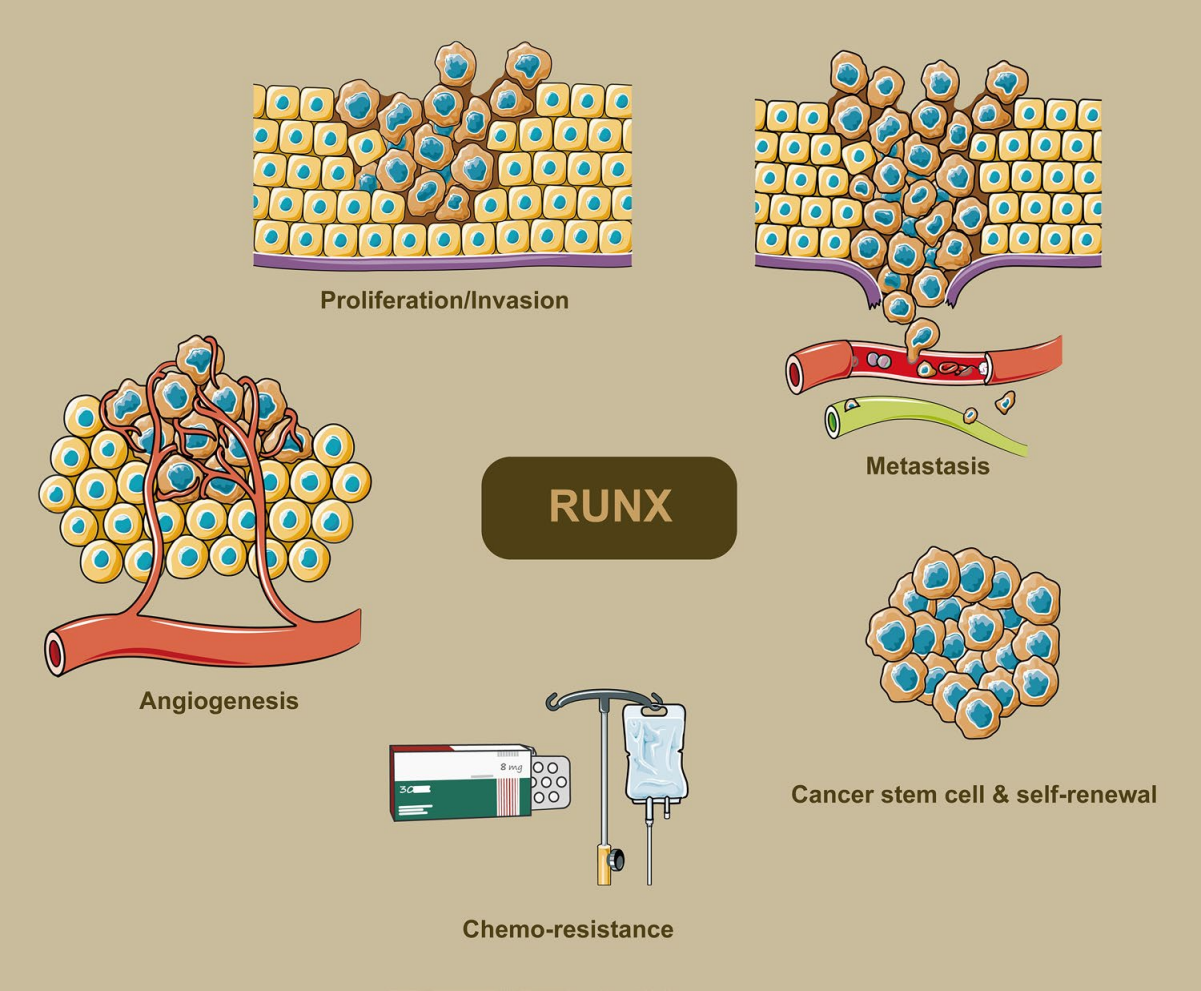
Comprehensive roles of RUNX family genes in oncogenesis. The diagram explicitly highlights the pivotal functions of RUNX genes across diverse cancer hallmarks, emphasizing the RUNX-associated effects on cellular proliferation and invasion, metastatic dissemination, angiogenesis, chemoresistance, and maintenance and self-renewal of cancer stem cells

RUNX's differential responses to oncogenic stimuli such as Wnt, c-Myc, and mutant RAS point towards its capacity for variable oncogenic activities. A core question that emerges is how RUNX effectively coordinates the crosstalk among multiple signaling pathways to integrate these signals and dictate cellular fate. Accumulating evidence implies that stringent regulation of RUNX expression is crucial for maintaining normal cellular differentiation. Disruption in this regulation could potentially lead to aberrant cellular differentiation, initiation of tumors, and subsequent tumor progression. Consequently, the expression levels of RUNX and its downstream targets could serve as early indicators of neoplastic development and as prognostic biomarkers. For a summarized overview of the differential roles and expression statuses of RUNX1, RUNX2, and RUNX3 in various cancers, readers are referred to Tables 1, 2, and 3. To encapsulate the complex roles and interactions of RUNX family genes in modulating the hallmarks of cancer, Fig. 3 serves as a representative scheme.

**Table 1:** Expression and oncogenic roles of RUNX1 in various human cancers

Cancer classification	expression status	Biological roles	Target genes and interacting proteins	References
ER-positive breast cancer	Mutations, implying downregulation	Tumor suppression through antagonizing oestrogen-mediated AXIN1 suppression	AXIN1, β-catenin	[41]
Triple negative breast cancer	Upregulated	Promotes cancer stem cell markers and chemotherapy resistance	Regulated by Androgen Receptor (AR)	[125]
Endometrial carcinoma	Upregulated	Inducer of distant metastasis, particularly to the lung	CBFβ and members of the Ets transcription factor family	[159]
Cervical cancer	Downregulated	Regulates the killing effect of NK cells on cervical cancer cells	Targeted by miR-20a; involved in the releases of IFN-γ and TNF-α	[44]
Cervical cancer	Upregulated	Involved in mediating EMT, promoting invasion and migration abilities	Upregulated by has-miR-616-5p and hsa-miR-766	[43]
Epithelial ovarian cancer	Upregulated	Promotes chemoresistance	Targeting IGF1R and cooperating with FOXO3a	[174]
Glioblastoma	Upregulated	Promotes migration, invasion, and angiogenesis	MMP-1, MMP-2, MMP-9, MMP-19, VEGFA	[17]
Glioblastoma multiform	Upregulated	Promotes temozolomide resistance	Negatively regulated by miR-128-3p; Upregulates MRP1	[50]
Glioblastoma, specifically the Mesenchymal subtype	Upregulated	Supports mesenchymal properties and promotes proneural-to-mesenchymal transition	Interacts with USP10 for stabilization; targeted by USP10 inhibitor Spautin-1	[49]
Pituitary tumors	Upregulated	Upregulates Galectin-3; potentially contributes to pituitary tumor progression	Galectin-3 (LGALS3)	[52]
Melanoma	Upregulated	Modulates resistance to BRAF-V600E inhibition	CSF-1R, IL-34	[172]
Neuroblastoma	Upregulated in well-differentiated tissues; downregulated in poorly differentiated tissues	Promotes apoptosis; inhibits metastasis and angiogenesis	Directly binds to promoters of BIRC5, CSF2RB, and NFKBIA	[51]
Oral squamous cell carcinoma	Upregulated	Promotes cell proliferation, adhesion, and migration; Inhibits apoptosis	miR-199a-3p	[53]
Lung cancer	Upregulated	Promotes growth and progression	Binds to ACP5 promoter, influences ERK/MAPK axis	[54]
Lung adenocarcinoma	Downregulated	Tumor suppressor; its downregulation is associated with worse survival and tumor aggression	E2F1 and multiple E2F1 target genes	[99]
Clear cell renal cell carcinoma	Upregulated	Protumorigenic, associated with poorer clinical survival	STMN3, SERPINH1, EPHRIN signaling pathways	[103]
Prostate cancer	Upregulated	Promotes EMT phenotype, drives metastatic migration and invasion	MMP2, MMP9, Akt/MAPK signaling pathways	[46]
Esophageal squamous cell carcinoma	Modulated (Affected by lincRNA-uc002yug.2)	Affects CEBPα expression, promotes ESCC progression	lincRNA-uc002yug.2, CEBPα	[185]
Pancreatic ductal adenocarcinoma	Upregulated	Contributes to tumor growth and resistance to apoptosis	Interacts with CBFβ; epigenetic regulation of NOXA promoter	[56]
Pancreatic cancer	Not specified for RUNX1 (but RUNX1-IT1 is upregulated)	Reduces the cancer-promoting effect of RUNX1-IT1	Interacts with C-FOS gene promoter; acts in conjunction with RUNX1-IT1	[55]
Hepatocellular carcinoma	Upregulated	Tumor suppressor that inhibits cell proliferation and migration	Acts as a transcriptional repressor for VEGFA	[142]
Hepatocellular carcinoma	Upregulated	Facilitates proliferation, migration, and invasion	COL4A1, FAK-Src signaling	[58]
Gastric cancer	Downregulated	Inhibits migration, invasion, and cell cycle process	Modulated by circ_0027599 via sponging miR-21-5p	[57]
Colorectal cancer	Upregulated	Promotes proliferation and chemoresistance	ABCG2, promoting its expression	[15]
Colorectal cancer	Upregulated	Promotes metastasis and EMT	Targeting KIT promoter	[25]

**Table 2:** Expression and oncogenic roles of RUNX2 in various human cancers

Cancer classification	Expression status	Biological roles	Target genes and interacting proteins	References
Breast cancer	Upregulated	Promotes bone metastasis	ITGA5 (Integrin α5)	[164]
Triple negative breast cancer	Upregulated	Facilitates aggressiveness and chemoresistance	Targeting MMP1 promoter, activating its transcription	[28]
Breast cancer, specifically in Invasive lobular carcinoma	Upregulated	Maintains breast cancer stem cell fate	Regulates phospho-PR target genes, notably SLC37A2	[131]
Thyroid cancer, Breast cancer	Upregulated	Promotes aggressiveness and metastatic spreading	Controlled by BRD4 and c-JUN	[69]
Epithelial ovarian cancer	Upregulated	Involved in proliferation, migration, and invasion	Downstream target genes include osteopontin and FAK; regulated by PKD2 and PKD3	[70]
Epithelial ovarian cancer	Upregulated	Involved in tumor progression and related to prognosis	Directly targeted by miRNA-23b	[104]
Cervical cancer	Upregulated	Positively regulates proliferation, associated with poor prognosis	Regulated by miR-218-5p	[67]
Oral cancer	Upregulated	Promotes proliferation, invasion, metastasis and EMT	CXCR4, AKT, FOXA2	[68]
Head and neck squamous cell carcinoma	Upregulated	Promotes cell growth and proliferation	PTHLH; stimulates the expression of cell cycle regulators like CCNA2, CCNE2, and CDC25A	[107]
Choroidal melanoma	Upregulated	Promotes migration and invasion	Directly targeted by METTL14 via N6-methyladenosine modification; Involved in Wnt/β-catenin signaling	[61]
Chondrosarcoma	Upregulated	Involved in chondroblastic malignant transformation, potentially via VEGF up-regulation	Interacts with JNK/ERK MAPKs, c-Jun and c-Fos (AP-1); Involved in VEGF up-regulation	[146]
Osteosarcoma	Upregulated	Essential for maintaining tumor cell survival	Induces SOX9, activates MYC; interacts with Menin and JMJD1C	[64]
Osteosarcoma	Upregulated	Promotes lung metastasis	Osteopontin (OPN/SPP1)	[62]
Lung adenocarcinoma	Upregulated	Promotes EMT, stemness, and invasion of airway epithelial cells	Upstream regulator of galectin-3; affected by intracellular ROS	[132]
Neuroblastoma	Not specified (Repressed by 30 kPa ECM stiffness)	Regulation of angiogenesis via VEGF-A secretion	VEGF165, YAP, SRSF1	[150]
Thyroid carcinoma	Upregulated	Promotes metastasis	Galectin-3 (Gal-3), MMP2/9	[30]
Clear cell renal cell carcinoma	Upregulated	Promotes malignant proliferation and migration	Downregulates NOLC1; upregulated by Zic2	[73]
Bladder urothelial cancer	Upregulated	Promotes EMT and extracellular matrix activities, linked to metastasis	associated with CAFs	[108]
Prostate cancer	Upregulated	Facilitates osteogenic differentiation of human bone marrow-derived mesenchymal stem cells (hBMSCs)	miR-205-5p/SFPQ/PTBP2 axis, upregulated by lncRNA NEAT1	[109]
Prostate cancer	Upregulated	Contributes to cell migration and tumor sphere formation	Forms a complex with CD44-ICD; regulates MMP-9 and osteopontin	[133]
Hepatocellular carcinoma	Upregulated	Promotes EMT, Vasculogenic Mimicry (VM), and invasion	Galectin-3 (LGALS3)	[110]
Pancreatic cancer	Upregulated	Contributes to resistance to gemcitabine (GEM) chemotherapy	TAp63, p53, ATM	[31]
Gastric cancer	Upregulated	Involved in metastasis; positively correlated with poor clinical outcomes	YAP1	[75]
Colon cancer	Upregulated	Epigenetic regulator of EMT, promoting metastasis and poor survival	EMT-associated genes	[112]
Colorectal cancer	Not specified	Sustains stem cell-like properties; promotes CD44-induced EMT; involved in invasion	Targets CD44; interacts with BRG1	[134]

**Table 3:** Expression and oncogenic roles of RUNX3 in various human cancers

Cancer classification	Expression status	Biological roles	Target genes and interacting Proteins	References
Breast cancer	Downregulated	Tumor suppressor; Inhibits estrogen-dependent proliferation	Targets estrogen receptor α (ERα), reducing its stability	[36]
Triple negative breast cancer	Downregulated	Inhibits migration and invasion	Targeted by miR-20a-5p; Direct downstream targets include Bim and p21	[80]
Epithelial ovarian cancer	Upregulated	Contributes to carboplatin resistance	Cellular inhibitor of apoptosis protein-2 (cIAP2)	[182]
High-grade serous ovarian cancer	Not specified	Influences platinum sensitivity and angiogenesis	BRCA1, γH2AX, Pt–DNA adducts, thrombospondin1	[155]
Glioblastoma	Upregulated (when LMTK2 is overexpressed)	Tumor inhibition, constrains Notch signaling	Notch signaling pathway	[116]
Cutaneous melanoma	Downregulated (in both primary and metastatic tumors)	Tumor suppressor, associated with overall survival	miR-532-5p	[79]
Melanoma	Downregulated	Inhibits cell migration and metastasis; induces cell shape change	MAL gene, genes related to adhesion and the actin cytoskeleton	[169]
Neuroblastoma	Downregulated	Tumor suppressor, constrains MYCN signaling; Low expression correlates with poor survival	Facilitates MYCN protein degradation and inhibits its downstream signaling	[114]
Papillary thyroid cancer	Downregulated	RUNX3 site-specific hypermethylation may offer value in predicting or monitoring postoperative recurrence of PTC patients	Methylation of RUNX3 at CpG sites -1397, -1406, -1415, and -1417	[113]
Lung adenocarcinoma	Downregulated	Tumor suppressor that inhibits alveolar hyperplasia	SP-B, CC10, Bmi1	[35]
Lung adenocarcinoma	Downregulated (implied, as it is targeted by miR-1275 which is upregulated)	Antagonist of Wnt/β-catenin and Notch signaling pathways, thereby inhibiting tumor stemness and metastasis	Interacts with miR-1275	[135]
Non-small cell lung cancer	Downregulated	Tumor suppressor; regulates bone resorption through chemokine modulation	CCL5, CCL19, CXCL11	[82]
Squamous cell carcinoma	Downregulated	Suppresses cancer stem cell phenotype	Interacts upstream with EZH2	[139]
Oral squamous cell carcinoma	Downregulated	Tumor suppressor; inhibits invasion and angiogenesis	Downregulates MMP-9; inhibits VEGF activity	[78]
Renal cell carcinoma	Downregulated	Inhibition associated with metastasis	CpG methylation, TGF-β	[85]
Prostate cancer	Downregulated	Inhibits metastasis and angiogenesis	TIMP-2, MMP-2, VEGF	[151]
Gallbladder cancer	Downregulated	Induction of ferroptosis in GBC cells	Activates ING1 transcription, represses SLC7A11 in a p53-dependent manner	[89]
Pancreatic ductal adenocarcinoma	Variable	Balances cancer cell division and dissemination, orchestrating a metastatic program	Dpc4/Smad4, Kras(G12D/+), Trp53(R172H/+)	[170]
Pancreatic cancer	Downregulated	Tumor suppressor; affects gemcitabine resistance	MRP1, MRP2, MRP5	[88]
Hepatocellular carcinoma	Downregulated	Reverses the oncogenic effects of miR-106b-5p	Direct target gene of miR-106b-5p	[90]
Esophageal cancer	Upregulated	functioning both as an oncogene and a tumor suppressor, influenced by interactions with MYC or p53	Co-expressed with EZH2, implicated in TGF-β dependent apoptosis	[86]
Gastric cancer	Downregulated	Resistance to growth-inhibitory and apoptosis-inducing action of TGF-beta	TGF-beta, R122C	[33]
Gastric cancer	Downregulated	Inhibits cell proliferation and peritoneal metastases	vav3, TOLL-like receptor, caspase 9	[91]
Gastric cancer	Downregulated	Inhibition of metastasis and angiogenesis	VEGF	[152]
Colorectal cancer	Downregulated	Suppresses metastasis and stemness	GLI1, promoting its ubiquitination	[13]
Colorectal cancer	Downregulated	Inhibits CRC cell proliferation and metastasis	Transcriptionally activates circMETTL3; circMETTL3 sponges miR-107, which targets PER3	[94]

Particularly intriguing is the question of whether the oncogenic propensity of RUNX can be mitigated by enhancing its oncogenic activity. This line of inquiry could illuminate if the restoration of RUNX expression represents a viable therapeutic strategy for cancer treatment. For instance, RUNX1's role in maintaining tumor cell stemness might be counteracted by the restored expression levels of RUNX3. In essence, it appears plausible that individual RUNX family members could act to mitigate the tumor-promoting effects of their counterparts.

The burgeoning field of research focused on the RUNX family of transcription factors holds considerable promise. As the field continues to expand rapidly, it is expected that our understanding of RUNX's pleiotropic roles in cancer therapeutics will become increasingly nuanced in the years to come. Unquestionably, in-depth and broad-based research is imperative and is likely to yield novel avenues for the development of anti-cancer pharmaceuticals.

## Data Availability

This is a review article that synthesizes existing findings, thus data availability is not applicable.
